# Using Real-Time fMRI Neurofeedback to Modulate M1-Cerebellum Connectivity

**DOI:** 10.1155/2022/8744982

**Published:** 2022-08-30

**Authors:** Yahia Madkhali, Salim Al-Wasity, Norah Aldehmi, Frank Pollick

**Affiliations:** ^1^Faculty of Applied Medical Sciences, Jazan University, Jazan, Saudi Arabia; ^2^College of Medical, Veterinary and Life Sciences (MVLS), University of Glasgow, Glassgow, UK; ^3^School of Psychology, University of Glasgow, Glassgow, UK

## Abstract

**Objective:**

The potential of neurofeedback to alter the M1-cerebellum connectivity was explored using motor imagery-based rt-fMRI. These regions were chosen due to their importance in motor performance and motor rehabilitation.

**Methods:**

Four right-handed individuals were recruited to examine the potential to change the M1-cerebellum neurofeedback link. The University of Glasgow Cognitive Neuroimaging Centre used a 3T MRI scanner from January 2019 to January 2020 to conduct this prospective study. Everyone participated in each fMRI session, which included six NF training runs. Participants were instructed to imagine complicated hand motions during the NF training to raise a thermometer bar's height. To contrast the correlation coefficients between the initial and last NF runs, a *t*-test was performed post hoc.

**Results:**

The neurofeedback connection between M1 and the cerebellum was strengthened in each participant. Motor imagery strategy was a significant task in training M1-cerebellum connectivity as participants used it successfully to enhance the activation level between these regions during M1-cerebellum modulation using real-time fMRI. The *t*-test and linear regression, on the other hand, showed this increase to be insignificant.

**Conclusion:**

A novel technique to manipulate M1-cerebellum connectivity was discovered using real-time fMRI NF. This study showed that each participant's neurofeedback connectivity between M1 and cerebellum was enhanced. This increase, on the other hand, was insignificant statistically. The results showed that the connectivity between both areas increased positively. Through the integration of fMRI and neurofeedback, M1-cerebellum connectivity can be positively affected.

## 1. Introduction

Cerebellar functions have received a lot of attention over the previous decades. The timing and accuracy of skilled motions rely on the cerebellum [1]. Functional neuroimaging in humans has revealed that motor learning and performance are linked to the cerebellum. The right motor cortex is linked to the left cerebellar hemisphere. If the left side of the cerebellum is damaged, the right side will be impacted. The cerebellum activation included a range of sensory and motor activities, according to previous functional magnetic resonance imaging (fMRI) studies. The cerebellum helps with several cognitive processes, such as motor imagery [2]. Several functional imaging studies have been conducted on the cerebellum. The first functional study of the cerebellum was conducted by Fox and colleagues [3]. They discovered that voluntary finger movement-activated bilateral lobule V. During a finger-tapping exercise, Kuhtz-Buschbeck, and colleagues [2] saw activity in the anterior cerebellum using fMRI.

The cerebellar regions that execute motor activities are different from those that execute cognitive and non-motor activities, according to Stoodley and Schmahmann [4]. Motor functions are controlled by the anterior cerebellum, while the posterior cerebellum controls cognitive functions. Since it aids in the pathophysiology of motor disorders linked to the cerebellum, understanding how cortical regions connect is critical. The cerebellum and the primary motor cortex (M1) were indirectly linked, according to Hoover and Strick [5], via MRI investigations. Daskalakis et al. [6], however, have proposed an indirect cross-link via the inferior parietal lobe; thus a direct connection to M1 is unlikely. Additionally, the cerebellum may be less directly connected with other cortical regions than that of M1, according to Manto et al. [7]. Many research such as functional connectivity in autism [8] and Parkinson's disease [9] have been conducted to investigate the link between the motor cortex and cerebellum. Ramos and colleagues [10] also utilized resting-state fMRI to investigate alterations in intrinsic cortico-cerebellar functional linkages in individuals with typical development. As a result of their research, Mostofsky and his colleagues examined youngsters with typical development (TD) and high-functioning autism (HFA) [11].

As a consequence of this investigation, the contralateral thalamus, ipsilateral cerebellum, contralateral main sensory cortex, and SMA were all engaged in motor execution. The contralateral thalamus, ipsilateral cerebellum, contralateral main sensory cortex, and SMA were all engaged in motor execution. The study found that the level of engagement was specific to the movement being executed, for example, when executing a hand gesture with the left hand, these regions were more active than when executing the same hand gesture with the right hand. This suggests that these regions are specifically activated during certain tasks or movements and may be important for their specific function. Moreover, these processes were demonstrated to be differentially engaged between TD children and ASD individuals. As a result of their investigation, Ramos et [10] suggested: “It might also help in targeting neuroimaging investigations that new treatment strategies eventually fade out as older or less potent drugs are substituted for them.” The authors further explored the specific areas of distributed abnormalities that comprised this fMRI activity in each situation.

Individuals may internally replicate actions or movements using Motor Imagery (MI) technique. Individuals may imagine about achieving motor things, but no physical outcomes are allowed in MI [11]. MI comes in two flavors: visual imagination (VI) and kinaesthetic imagination (KI) [11]. MI has a significant function since it is often utilized in motor learning exercises [12]. It may be beneficial to athletes, skill development, and rehabilitation since it may be used to improve motor performance over periods [13]. When physical movement, such as clenching or tapping, was imagined by participants using MI, the cerebellum was activated [10]. According to a study by Blefari et al. [12], even though cerebellar activity was high during motor execution, it decreased by 30% during MI. Neurofeedback is using visual or auditory stimuli to assist in self-regulation in a person [12]. Real-time fMRI (rt-fMRI) systems have been used for neurofeedback because of their speed and capacity to supply signal feedback from the brain activity of the subcortical brain framework. Neurofeedback has been shown to be an effective treatment for a wide range of disorders, including depression and anxiety. One potential mechanism by which neurofeedback may work is by modulating brain function. Previous studies have shown that motor imagery-based rt-fMRI connectivity is associated with cerebellum function. The current study aim is to determine whether neurofeedback using motor imagery-based rt-fMRI connectivity is able to modulate M1-cerebellum connectivity.

The current research aimed to see whether people's brain feedback connections between the primary motor cortex (M1) and cerebellum might be changed. These adjustments may provide insight into healthy people's link between the cerebellum and motor cortex. The M1-cerebellum coupling has never been studied in conjunction with rt-fMRI modulation. Modifying the M1-cerebellum connectivity may help in illness therapy such as Parkinson's disease and autism by improving motor functions [11, 16].

## 2. Methods

### 2.1. Participants

This study included four individuals (three men and one woman) with normal or corrected vision. According to the participants, all the material and photos on the screen were viewable. According to the Edinburgh Handedness Inventory [17], all participants were right-handed. English was a breeze for all participants, whether they spoke or wrote it. Participants' average age was 36 years old. Each participant completed the Vividness of Movement Imagery Questionnaire-2 (VMIQ-2) to assess their capacity to perform the motor imagination tasks. The VMIQ-2[18, 19] is a 12-question exam that measures an individual's ability to generate new motions. There are two types of motor imagery: internal (first person) and external (third person) visual imagery. As seen in Table 1, the VMIQ-2 has passed all three types of validity tests: factorial, construct, and concurrent.

This study received ethical approval from the College of Science and Engineering ethics committees at the University of Glasgow. Each participant approved the experiment. Before undergoing the MRI scanning, each participant received a thorough explanation of the experiment's complete technique; all participants completed an MRI safety checklist.

### 2.2. Imaging Parameters and Rt-fMRI Neurofeedback Platform

Between January 2019 and January 2020, the University of Glasgow Centre for Cognitive Neuroimaging (CCNi) conducted this prospective study using a 3T Siemens Tim Trio MRI scanner with a 32-channel head coil. The Echo Planar Imaging (EPI) technique (TR = 2000 ms, 0.3 mm gap, 32 axial slices, TE = 30 ms) was used to acquire *T*2 -weighted functional scans. All participants in this study underwent a high-resolution anatomical scan (*T*1 weighted image), six NF runs, and a functional localizer run. The scanner provided Turbo-BrainVoyager (TVB) software with useful data over a network connection during NF runs. Preprocessing of the functional data took place in real-time. The correlation between the two regions was calculated using custom MATLAB code, which was then displayed as a thermometer bar on the computer. During their NF training, the participants were encouraged to imagine complex hand motions to raise the height of the thermometer bar. For the motor imagery task, there were no particular hand motions recommended. Participants were able to test different mental strategies and actions to raise their thermometer. The height of the thermometer bar was determined using the Pearson correlation coefficient. The thermometer bar level was updated and displayed after each NF block using an intermittent feedback paradigm. During the localizer run, 7 fixation blocks (16 s) intermingled with 6 bimanual hand clenches (the 30 s). Participants were supposed to count letters or numbers when “REST” appeared and clench their fists when “Clench” occurred during functional scanning (the localizer). The block labeled “Clench” lasts 30 seconds, whereas the block labeled “Rest” lasts 16. The available data were processed and analyzed online using BrainVoyager. Each participant's original space was used to generate the ROIs.

### 2.3. Rt-fMRI Neurofeedback

**Figure 1:**
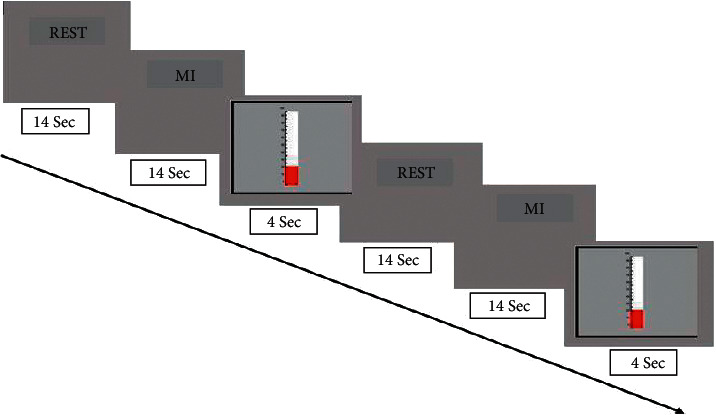
The NF training paradigm is constructed using fMRI. When the thermometer bar was shown for 4 seconds, a run lasted 366 seconds and included twelve 14 s fixations (rest) blocks and eleven 14 s NF blocks.

Every participant completed 366-second NF training runs. Every NF training run included 32 s blocks. Each cycle consisted of a 14-second break (twice repeated throughout each cycle since each cycle began and ended with a rest), an 11-second MI exercise, and four seconds of feedback signal (Figure 1). During the blocks, participants were told to perform imagination of complicated motions for 14 seconds to raise the activation level between the M1-cerebellum connection. After each NF block, the thermometer bar displayed M1-cerebellum integration for 4 seconds. Participants were instructed to count numbers or letters for 14 seconds to keep track of their baseline activity [17].

### 2.4. Online Data Analysis

Turbo-BrainVoyager software and MATLAB were used to analyze and present rt-fMRI data. A network link was used to transfer the data to Turbo-BrainVoyager software. In real-time, the available data were preprocessed. To update and display the feedback signal, which included an intermittent thermometer, the following equation was used:

(1) $$\begin{array}{c} bar\;height=\text{scale}*\left(\text{Corr}+1\right). \end{array}$$

To improve the visibility of the thermometer bar, the scale is an arbitrary scale value.

(2) $$\begin{array}{c} \frac{\sum_{i=1}^{n}\left(x_{i}-\bar{x}\right)\left(y_{i}-\bar{y}\right)}{\sqrt{\sum_{i=1}^{n}{\left(x_{i}-\bar{x}\right)}^{2}}\sqrt{\sum_{i=1}^{n}{\left(y_{i}-\bar{y}\right)}^{2}}}. \end{array}$$

The mean activation of *M*1 during a MI block is represented by *x*, which is a 14-s time course of M1, and the mean activation of the cerebellum is represented by *y*.

### 2.5. Full Brain Analysis

BrainVoyager QX 2.8.4 (Brain Innovation, Maastricht, The Netherlands) was used to manipulate the available data, which was then imported into MATLAB. Before being normalized to Talairach space, inhomogeneity was corrected in the anatomical *T*1 weighted image. Moreover, all volumes were spatially aligned to the localizer run's first volume to eliminate head motion, and the functional data were slice-time corrected. After that, Brain Voyager was used to align the fMRI data to *T*1 weighted image using anatomical landmark points if necessary. A rigid body transform and scaling were used to convert the data to Talairach space. Nonlinear drifts in the time series were eliminated using a temporal high-pass filter (2 cycles) applied to linear drifts. A Gaussian kernel with a 6-mm full width at half maximum was used to smooth the functional data. To analyze preprocessed functional data with two predictors (fist clenching and rest) and six head motion parameters, a General Linear Model (GLM) was utilized for each subject [18]. A second-level random-effect analysis general linear model (RFX-GLM) was used to test group data. The generated statistical maps were thresholded and corrected for multiple comparisons using cluster-level thresholding [19]. The blocks of the NF were then contrasted to the baseline (*p* < 0.01 corrected for cluster-level thresholding) in all six NF runs.

### 2.6. Statistical Analysis

A *t*-test was used as a post hoc test to examine the correlation between the first and last runs. Linear regression was also performed using the average connectivity values of each neurofeedback run in order to calculate the upregulation over NF runs.

## 3. Results

**Figure 2:**
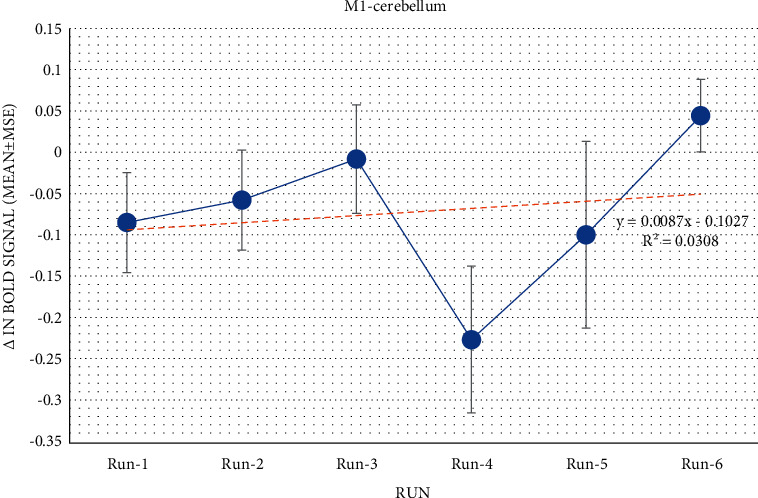
When comparing the first and last runs, this statistic shows the average BOLD signal change in *M*1-cerebellum connectivity. The standard deviation of the mean represented the error bars.

The participants' cerebellar and M1 activation was observed during the localizer, owing to hand clenching. Each participant completed all six runs successfully. Participants seemed to upregulate activation between M1 and the cerebellum before dropping at run 4.  Figure 2 shows an upwards trend in activation levels across runs.

Nonetheless, participants showed a trend for successfully upregulating based on the linear regression results (Intercept coefficient = −0.103, *R*^2^ = 0.272 and *p* value = 0.0739). These findings suggested effective modulation due to differences in activation levels from run 1 to run 6.

According to the paired *t*-test, the observed increase in correlation was not statistically significant (*p* = 0.287). As result, four participants were not able to upregulated the neurofeedback connectivity between M1 and the cerebellum using real-time fMRI.

**Figure 3:**
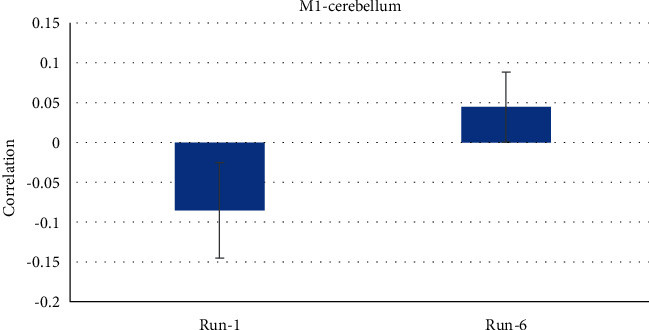
This graph shows the connectivity between *M*1 and the cerebellum in the first and final runs. The standard deviation of the mean is represented by the error bars.

In addition, participants thought playing music, knocking on doors, or lifting weights might help them raise their thermometer bar as shown in Figure 3.

### 3.1. Whole Brain Analysis

**Figure 4:**
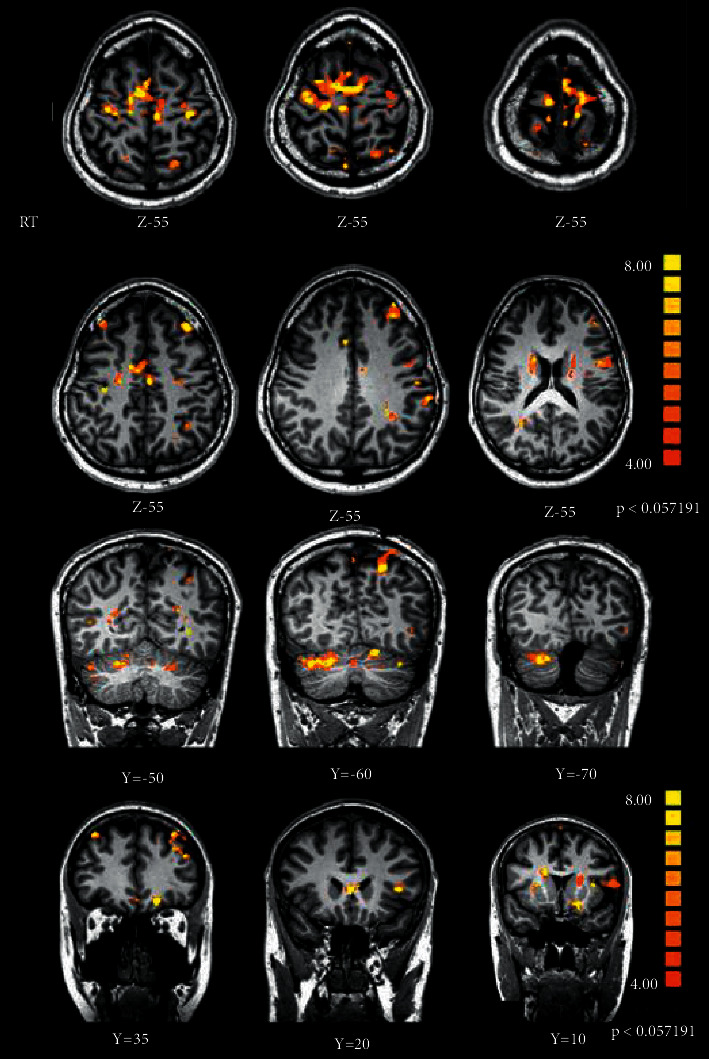
For the NF group (coronal images), the results of the NF runs analysis are displayed. At *p* < 0.01 (uncorrected), these activations are significant.

To see if any brain areas were activated during NF-directed motor imagery, a comprehensive RFX-GLM analysis (*p* < 0.01 corrected) was performed. Whole brain analysis shows that activations were seen in the cerebellum and M1 where the feedback was received. Also, bilateral parietal lobes, bilateral insula, and caudate body were all activated.  Table 2 and Figure 4 list and illustrate the activated areas during this real-time fMRI experiment.

## 4. Discussion

In a single session of motor imagery of complex body motions with intermittent feedback signals (represented as the height of a thermometer bar) using real-time fMRI, we discovered preliminary evidence that healthy people might learn to increase their activity level between *M*1 and cerebellum connectivity. To our knowledge, real-time fMRI has never been used to investigate the relationship between cortical motor regions and the cerebellum previously. Despite this, studies investigating *M*1-cerebellum connectivity have been conducted using resting-state fMRI [6]. Attempts to conclude the efficacy of modulation between cortical and cerebellum are hampered by the limited number of studies that have been conducted [20]. In this research, participants had the potential to upregulate the activity level between M1-cerebellum connectivity since the correlation in the last run was stronger than that of the first. A linear regression of average connectivity values was utilized to measure the upregulation over neurofeedback runs.

The results indicate that *M*1-cerebellar connectivity seems to be increased by time. However, *t*-test indicates that the upregulating is not statistically significant. Furthermore, through linear regression, the NF group's mean *M*1-cerebellum activity was not apparent. This means that there was no significant relationship between NF and M1-cerebellum activity. In other words, the findings suggest that being NF does not seem to have an impact on cerebellum activity. This suggests that increased *M*1-cerebellum connectivity was likely a product of neurofeedback modulation and not intrinsic changes made by participants after putting more effort to enhance the modulation.

This study has several implications for clinicians and scientists who are investigating interactions between brain function and mental disorders. First, it suggests that further research is needed in this area before any firm conclusions can be drawn about whether or not being NF affects cerebellar function. Second, it highlights the importance of using appropriate methodology when studying these types of relationships—particularly when looking at *M*1-cerebellum activation levels.

A *t*-test was used as an exploratory examination to contrast the correlation between the first and final runs. Despite this, neither test resulted in a statistically significant result. This study recruited four participants at a time (four). This study's impact is modest because bigger sample sizes result in more effects. Motor imagery (MI) is a condition in which a person performs mental motor tasks but has no physical motor output [21]. According to several prior studies, MI seems to activate comparable motor areas as those engaged in preparation for and actual execution of actions [22]. Mental tasks in motor imagery have modulated brain activity in specific areas such as *M*1 [23] and SMA [24].

The study by [12, 14] showed that cerebellar activation and M1 activation resulted from motor imagery tasks. These findings are consistent with those of our research because they showed cerebellar activation and *M*1 activation resulting from the same type of task. This is an important finding because it supports the notion that the brain responds similarly to different types of mental activities. The authors note that this could be due to the encoding of movement representations within cerebellum and muscle cortex, respectively. This may allow for improved coordination or speed during movements downstream from these areas. Additionally, the increased cortical activity might account for the subjective experience of imaging motor actions while performing the experiment.

In this rt-fMRI NF research, the implicit strategy is often employed in which participants were instructed to search for a strategy that enhances their activation score during the experiment [25]. Participants reported that actions such as lifting weights or playing music increased the thermometer bar during the neurofeedback experiment. However, the downside of the implicit strategy is participants may quit exploring new mental actions if they discover one that boosts their neural activity level [25].

Whole-brain activity and brain-distributed learning processes can be monitored and investigated using real-time fMRI. Regarding most real-time fMRI investigations, the training effects are mostly seen in the neurofeedback target region [23]. This may be because this is where the focus of attention typically lies during cognitive tasks, such as those used in neurofeedback training. It has been shown that repeated exposures to a stimuli or task over time can lead to an enhanced response within that particular region of the brain. However, some investigations report increased activation throughout the whole brain during neurofeedback training, which might be due to general enhancements of cognitive function that result from neurofeedback training or specific neural changes induced by neurofeedback stimulation in certain regions. Future research should explore this question more closely and determine which regions of the brain may benefit from neurofeedback intervention most specifically. The influence of self-regulation and learning of self-regulatory strategies on a successful connection has been investigated [29]. According to numerous real-time fMRI NF research, neurofeedback training affects distinct changes in target region connectivity, predominantly strengthening important connections while weakening others. Consequently, whole-brain analysis permits researchers to see how activation and network alterations occur in specific regions. The full-brain examination was used during the training process to see how the brain became activated.

Bilateral parietal lobe activation was seen during NF training. The processing of visual stimuli could have triggered these areas. Participants got visual feedback from a thermometer bar to show the activation levels of a motor imagery task throughout the experiment. These feedback projections may boost parietal lobe recruitment via real-time neurofeedback [30]. There has been a lot of recent research exploring how different types of imagery (e.g., visual, auditory, olfactory, etc.), and in particular motor imagery, can influence various aspects of cognitive function.

In a recent study, researchers looked at how motor imagery might be related to brain activation. They hypothesized that using motor imagery (MI) might be linked to fMRI activation in the left parietal lobe [31, 32]. The insula may be activated during NF training because it processes intentional actions [33]. Furthermore, it's possible that using motor imagery (MI) is linked to fMRI activation in the left parietal lobe. The insula may be activated during NF training because it processes intentional movement and awareness [36]. In addition, activation in the sensorimotor-related brain regions, such as the insula, may be linked to self-agency perception during voluntary motor activities [32, 36]. The insula is also a critical component of the brain, as Emmert et al. [36] demonstrated during real-time fMRI neurofeedback.

Real-time fMRI NF may alter M1-cerebellum connectivity, according to current findings. The complete-brain analysis's activation in the whole brain and the cerebellum bolstered this conclusion. Lindeman and colleagues were able to modify M1 and the cerebellum using a non-MRI approach. They activated Purkinje neurons using whiskers to alter the coherence between S1 and M1. We recommend doing more research with a bigger sample size in the future because our online and offline findings suggest an impact on targeted connectivity [37].

## 5. Conclusion

Neurofeedback is a new and advance method that has been found to help people with various mental health conditions, such as anxiety and depression. Neurofeedback is based on the theory that your brain functions in much the same way as a computer—you can train it to respond in a certain way by providing feedback to it. By using neurofeedback, patients can learn how to control their motor, thoughts, and emotions and improve their overall mental health and motor outcome.

The cerebellum plays an important role in skilled movement timing and precision. M1 is critical in addition to planning and carrying out actions. The possibility of changing the M1-cerebellar connection was highlighted using real-time fMRI. We used this method in our research and found that each participant's neurofeedback connection between M1 and the cerebellum appeared to strengthen. Nevertheless, this improvement was not statistically significant. Further investigation, the recruitment of additional participants, and forming a control group are advised.

## Figures and Tables

**Table 1:** Types of validity tests: factorial, construct, and concurrent.

	M1-cerebellum (mean)
Age (years)	36
Handedness	92.5
MI vividness (third-person perspective)	22.75
MI vividness (first-person perspective)	21.5

**Table 2:** Clusters of brain activations seen during the NF modulation.

Cortex	*X*	*Y*	*Z*	*t*	*P*-value	Number of voxels
RT parietal lobe, BA 7	21	−58	52	23.923	0.00174	5502
RT insula, BA 13	32	−22	13	109.41	0.00008	14583
RT caudate body	12	7	25	52.201	0.00037	1470
LT parietal lobe, BA 7	−21	−58	34	54.443	0.000337	1278
LT precentral gyrus, BA 4	−33	−19	55	17.8134	0.003137	257
LT insula, BA 13	−33	8	10	92.853	0.000116	3903
LT frontal lobe, BA 9	−39	47	28	47.463	0.000444	2581
LT cerebellum, anterior lobe	−32	−58	−23	23.6665	0.00178	646
RT cerebellum, posterior lobe	33	−64	−24	9.0189	0.002878	308
RT medial frontal gyrus, BA 6	15	−7	56	24.7062	0.000145	2221
LT superior frontal gyrus, BA 6	−9	−1	63	7.4269	0.00505	603
RT cerebellum, anterior lobe	21	−59	−23	23.9235	0.00174	5502
LT cerebellum, posterior lobe	−12	−59	−14	31.8582	0.00098	520

## Data Availability

The data used to support the findings of this study are included within the article.
